# Supplementary use of HbA1c as hyperglycemic criterion to detect metabolic syndrome

**DOI:** 10.1186/1758-5996-6-119

**Published:** 2014-11-06

**Authors:** Parco M Siu, Queenie S Yuen

**Affiliations:** Department of Health Technology and Informatics, Faculty of Health and Social Sciences, The Hong Kong Polytechnic University, Hung Hom, Hong Kong China

## Abstract

**Background:**

Metabolic syndrome (MetS) refers to a cluster of cardiovascular risk factors including hyperglycemia, dyslipidemia, abdominal obesity and hypertension. An effective detection of MetS not only reflects the prediction risk of diabetes mellitus and cardiovascular diseases but also helps to plan for management strategy which could reduce the healthcare burden of the society. This study aimed to compare the use of hemoglobin A1c (HbA1c) to fasting plasma glucose (FPG) as the hyperglycemic component in MetS diagnosis.

**Methods:**

Waist circumference, blood pressure, blood triglyceride, high-density lipoprotein (HDL)-cholesterol, FPG, and HbA1c were examined in 120 Hong Kong Chinese adults with MetS and 120 without MetS. After reviewing the subject basal characteristics, 11 of them were found with undiagnosed diabetes (FPG ≧7.0 mmol/L) and were excluded for further analysis.

**Results:**

The most prevalent MetS components among the included subjects were elevated systolic blood pressure and central obesity. Significant correlation relationships existed between FPG and HbA1c in both subject pools diagnosed with and without MetS (p < 0.001). The diagnostic rate of MetS using HbA1c was compared to FPG by the receiver operating characteristics (ROC) analysis which suggested an area under curve of 0.807 (95% CI: 0.727 to 0.887). The agreement was 90.7% in MetS-positive group with increased FPG as one of the criterion co-existed with elevated HbA1c. If including HbA1c as an additional criterion to FPG in the MetS diagnosis, 30 more participants in MetS-negative group would be MetS-positive leading to an increase in detection rate. Furthermore, 47 subjects (38 from MetS-positive group and 9 from MetS-negative group) were found having HbA1c ≧6.5%, who would have been diagnosed with diabetes based on the diagnostic criteria implemented by the Expert Group in 2009.

**Conclusion:**

These findings suggest that HbA1c enhances the detection of hyperglycemia for the diagnosis of MetS.

## Background

Metabolic syndrome (MetS) refers to a cluster of metabolic abnormalities including hyperglycemia, dyslipidemia, abdominal obesity and high blood pressure, which is closely associated with the development of type 2 diabetes mellitus and cardiovascular diseases. The incidence of diabetes and cardiovascular diseases has been reported to be considerably increased in individuals with MetS [1, 2]. The risks of having heart disease, stroke, and diabetes have been shown to be increased by 1.5- to 3-fold in people with MetS when compared to people without MetS [3].

The occurrence of MetS is suggested to be affected by many factors including genetics, stress, improper diet, lack of physical activity, weight gain, increased alcohol consumption, smoking, and inadequate sleep [4–10]. The prevalence of MetS worldwide is approximately 20-30% in general population and it is apparently on an increasing trend. In the United States, the prevalence was 23.7% which was similar to Europe and the percentage appeared to be higher in population with advanced age [11]. In Asia, the prevalence of MetS reported in different countries were quite varied ranged from 21.9% in Thailand to 49.4% in Malaysia [11, 12]. The prevalence of MetS in Korea has been shown to be significantly increased from 24.9% in 1998 to 31.3% in 2007 [12]. The prevalence of MetS in mainland China and Hong Kong has been reported to be 29.3% in middle-aged Chinese men and 26.8% in Hong Kong professional drivers [10, 13]. A cohort study has demonstrated that the crude percentage of MetS had indeed been increased from 9.6% in 1990s to 23% in 2000s in Hong Kong [14]. These figures indisputably illustrated that MetS has been a global alarming public health concern [15].

Intervention applied to individual with MetS offers an early effective approach to prevent or delay the onset of the associated chronic diseases including diabetes and cardiovascular diseases. From a public health point of view, appropriate management of MetS through lifestyle modification such as dietary management and physical exercise has a significant healthcare value to combat with the growing prevalence of diabetes and cardiovascular diseases [1, 5, 8–10, 16, 17]. Successful reversal of the MetS conditions should have considerable impact to diminish the number of emerging cases of diabetes and cardiovascular diseases, which would largely lessen the future healthcare expenses associated with diabetes and cardiovascular diseases [18]. Thus, effective detection of MetS not only reflects the prediction risk of diabetes and cardiovascular diseases but also helps to plan for management strategy which could reduce the healthcare burden of the society. In this study, we aimed to examine the supplementary use of hemoglobin A1c (HbA1c) as the hyperglycemic component for MetS diagnosis. HbA1c was examined because of its higher reproducibility and lower biological variation relative to fasting plasma glucose (FPG).

## Methods

### Subjects

In this case-control study, 240 Hong Kong Chinese adults (120 diagnosed with MetS and 120 without MetS) aged 30 to 80 years from the community were voluntarily recruited. Informed consent was obtained from all subjects. All the experimental procedures received human research ethics approval from The Hong Kong Polytechnic University.

### MetS parameters and blood biochemistry

Cardiovascular measurements including systolic and diastolic blood pressure were examined by an electronic blood pressure monitor (Accutorr Plus, Datascope). Blood pressure measurement was determined on the right arm after 5-min seated rest. Systolic and diastolic blood pressure was obtained over the brachial artery region with the arm supported at heart level using appropriate sized cuff. The average of two measurements taken with a 1-min interval between them was recorded for analysis. Waist circumference was measured midway between the lowest rib and the superior border of the iliac crest using an inelastic measuring tape on the bare skin and recorded to the nearest 0.1 cm. The tape was snugged horizontally around the abdomen passing across the navel without causing compression on the skin. Measurement was performed at the end of normal expiration. Biochemical measurements were performed on fasting plasma harvested from venous blood samples collected after an overnight (a minimum of 10 hours) fast. Plasma glucose, triglycerides, and HDL-cholesterol concentrations were measured by an accredited medical laboratory by commercial test kit methods using an automatic clinical chemistry analyzer (Architect CI8200, Abbott Diagnostics).

### Definition of Metabolic Syndrome (MetS)

Metabolic syndrome (MetS) was diagnosed according to the guideline of the United States National Cholesterol Education Program (NCEP) Expert Panel Adult Treatment Panel (ATP) III criteria, in which an individual diagnosed with MetS has three or more of following characteristics: 1) central obesity (waist circumference exceeds 90 cm or 80 cm for Asian male and female, respectively), 2) hypertension (systolic pressure equals or exceed 130 mmHg or diastolic pressure equals or exceeds 85 mmHg), 3) elevated blood glucose (fasting glucose level equals or exceeds 5.6 mmol/L [100 mg/dL]), 4) elevated plasma triglycerides (level equals or exceeds 1.70 mmol/L [150 mg/dL]), and 5) low level of high-density lipoprotein-cholesterol (HDL-C; level equals or is less than 40 mg/dL for male and 50 mg/dL for female) [19].

### Measurement of hemoglobin A1c (HbA1c)

HbA1c was measured by ion exchange HPLC method using the VARIANT II Hemoglobin A1c Program manufactured by Bio-Rad Laboratories. The method is traceable to the reference methods of both the National Glycohemoglobin Standardization Program (NGSP) and the International Federation of Clinical Chemistry and Laboratory Medicine (IFCC). Also, it has been certified by the NGSP as having documented traceablility to the Diabetes Control and Complications Trial (DCCT) reference method (American Diabetes Association). The VARIANT II Hemoglobin A1c Program is based on the principles on ion exchange high performance liquid chromatography (HPLC) method by a programmed buffer gradient, of increasing ionic strength, to the cartridge where the hemoglobins are separated based on their specific ionic interactions with the cartridge material. The separated hemoglobin then passed through the flow cell of the filter photometer, where the changes in the absorbance at 415 nm were measured. An additional filter at 690 nm was used for background absorbance correction. A chromatogram was generated with the HbA1c peak shaded and the percentage of HbA1c was reported in SI percent units (HbA1c/total Hb). In order to convert the SI percent units to the IFCC SI units (mmol/mol) which was recommended in a consensus statement issued by the working group including the ADA, IDF, European Association for the Study of Diabetes and IFCC in 2007, the equation of IFCC: HbA1c(mmol/mol) = [HbA1c(%)–2.15] × 10.929 was adopted (American Diabetes Association). The analysis was performed in four consecutive days and each blood sample was analyzed twice and the mean results were obtained. The maximum within run percentage of coefficient of variation (%CV) using two lyophilized whole blood samples for non-diabetic range (4.7–5.9%) and diabetic range (8.7-11.1%) were 2.8% and 1.7%, respectively. The across run%CV using the same lyophilized whole blood samples were 3.6% and 3.8%, respectively.

### Data analysis

Statistical analyses were performed by using Statistical Package for Social Science (SPSS), version 17.0 for windows. Data are expressed as mean ± standard deviation. Independent t-test or Mann-Whitney U test were used to compare the difference between groups. The relationship between HbA1c and fasting plasma glucose (FPG) across groups was analyzed by Pearson correlation or Spearman correlation. P-value of <0.05 would be considered statistically significant. Chi-square by McNemar test was used to compare the use of HbA1c to FPG to detect MetS. The diagnostic property of using HbA1c and FPG for MetS was evaluated by receiver operating characteristics (ROC) curve with the 95% confidence intervals.

## Results

Among the 240 subjects examined, 120 were diagnosed with metabolic syndrome (MetS-positive) and 120 were not having metabolic syndrome (MetS-negative) based on the NCEP diagnostic guideline. Of note, 11 MetS-positive subjects (9 male and 2 female) were found having fast plasma glucose (FPG) ≧7 mmol/L, which were classified as diabetic according to the consensus statement in 2009 [20] and were thus excluded in this study. In sum, data obtained from 109 MetS-positive subjects (51 male and 58 female) and 120 MetS-negative subjects (60 male and 60 female) were included in our analyses. The basal characteristics of these 229 subjects are shown in Table 1.

**Table 1:** **Basal characteristics of the subjects**

	All n = 229	MetS-positive n = 109	MetS-negative n = 120	P-value
Age (years)	58.7 ± 10.5	59.1 ± 10.6	58.3 ± 10.4	0.556
WC (cm)	85.9 ± 10.6	92.8 ± 8.2	79.6 ± 8.3	<0.001
SBP (mmHg)	139.5 ± 20.3	145.3 ± 17.7	134.1 ± 21.1	<0.001
DBP (mmHg)	79.4 ± 11.1	82.5 ± 10.9	76.7 ± 10.6	<0.001
FPG (mmol/L)	5.3 ± 0.6	5.6 ± 0.6	5.0 ± 0.4	<0.001
TG (mmol/L)	1.5 ± 0.7	1.9 ± 0.7	1.2 ± 0.5	<0.001
HDL (mmol/L)	1.4 ± 0.3	1.2 ± 0.3	1.6 ± 0.3	<0.001
HbA1c (%)	6.1 ± 0.5	6.3 ± 0.6	5.9 ± 0.4	<0.001
Correlation coefficient between HbA1c and FPG	0.593, P < 0.001	0.569, P < 0.001	0.397, P < 0.001	

Note: SBP, systolic blood pressure; DBP, diastolic blood pressure; WC, waist circumference; HDL, high-density lipoprotein cholesterol; TG, triglyceride; FPG, fasting plasma glucose; HbA1c, haemoglobin A1c; data are expressed as mean ± standard deviation. Metabolic syndrome (MetS) was diagnosed according to the guideline of the United States National Cholesterol Education Program (NCEP) Expert Panel Adult Treatment Panel (ATP) III criteria, in which an individual diagnosed with MetS has three or more of following characteristics: 1) central obesity (waist circumference exceeds 90 cm or 80 cm for Asian male and female, respectively), 2) hypertension (systolic pressure equals or exceed 130 mmHg or diastolic pressure equals or exceeds 85 mmHg), 3) elevated blood glucose (fasting glucose level equals or exceeds 5.6 mmol/L [100 mg/dL]), 4) elevated plasma triglycerides (level equals or exceeds 1.70 mmol/L [150 mg/dL]), and 5) low level of high-density lipoprotein-cholesterol (HDL-C; level equals or is less than 40 mg/dL for male and 50 mg/dL for female) [19].

The prevalence of individual MetS components and HbA1c is summarized in Table 2. Among the 229 subjects, the most frequently met MetS criteria were systolic blood pressure (SBP) (n =161, 70.3%) and waist circumference (n =125, 54.6%) (Table 2). The prevalence of hyperglycemia defined as elevated FPG (defined as FPG ≧5.6 mmol/L [100 mg/dL]) was found in 65 subjects (28.4%) whereas elevated HbA1c (defined as HbA1c ≧5.7%) was found in 194 subjects (84.7%) (Table 2). This observation demonstrated that a large portion of our examined subjects (i.e., 84.7% - 28.4% =56.3%) had normal level of FPG but elevated level of HbA1c. If using HbA1c as a hyperglycemic criterion in MetS definition, the number of subjects with different number of components present when using FPG criterion or HbA1c criterion was summarized in Table 3. The percentage of MetS-positive subjects would be increased from 47.6% (109 out of 229 subjects) by using FPG as the criterion to 60.7% (139 out of 229 subjects) by using HbA1c as the criterion.

**Table 2:** **Prevalence of individual MetS components and HbA1c**

	All	MetS-positive	MetS-negative
Number (%)	229 (100%)	109 (100%)	120 (100%)
WC	125 (54.6%)	101 (92.7%)	24 (20.0%)
SBP	161 (70.3%)	94 (86.2%)	67 (55.8%)
DBP	112 (48.9%)	67 (61.5%)	45 (37.5%)
FPG	65 (28.4%)	59 (54.1%)	6 (5.0%)
TG	90 (39.3%)	68 (62.4%)	22 (18.3%)
HDL	70 (30.6%)	61 (56.0%)	9 (7.5%)
HbA1c	194 (84.7%)	99 (90.8%)	95 (79.2%)

Note: SBP, systolic blood pressure; DBP, diastolic blood pressure; WC, waist circumference; HDL, high-density lipoprotein cholesterol; TG, triglyceride; FPG, fasting plasma glucose; HbA1c, haemoglobin A1c. Data are expressed as frequency count (%). Metabolic syndrome (MetS) was diagnosed according to the guideline of the United States National Cholesterol Education Program (NCEP) Expert Panel Adult Treatment Panel (ATP) III criteria, in which an individual diagnosed with MetS has three or more of following characteristics: 1) WC exceeds 90 cm or 80 cm for Asian male and female, respectively, 2) SBP equals or exceed 130 mmHg or DBP equals or exceeds 85 mmHg, 3) FPG equals or exceeds 5.6 mmol/L [100 mg/dL], 4) TG equals or exceeds 1.70 mmol/L [150 mg/dL]), and 5) HDL-C equals or is less than 40 mg/dL for male and 50 mg/dL for female) [19]. Elevated HbA1c is defined as HbA1c ≧5.7%.

**Table 3:** **Number of subjects with different number of components present when using fasting plasma glucose (FPG) or HbA1c as the hyperglycemic criterion**

Number of MetS component present		0	1	2	3	4	5	Met ≧3 components (MetS-positive)
FPG as criterion	n	32	45	43	56	43	10	109
	(%)	(14.0)	(19.7)	(18.8)	(24.5)	(18.8)	(4.4)	
HbA1c as criterion	n	10	40	40	59	59	21	139
	(%)	(4.4)	(17.5)	(17.5)	(25.8)	(25.8)	(9.2)	

As shown in the cross-tabulation table between FPG and HbA1c (Table 4), only 55.7% of subjects (59/109) had FPG ≧5.6 mmol/L as one of the criterion in MetS-positive group. However, 90.8% of subjects (99/109) had HbA1c ≧5.7%, 91.7% of subjects (100/109) had either increased FPG or HbA1c, and 53.2% of subjects (58/109) had both increased FPG and HbA1c. In MetS-negative group, only 5% of subjects (6/120) had FPG ≧5.6 mmol/L but 79.2% of subjects (95/120) had HbA1c ≧5.7%, 80% of subjects (96/120) had either increased FPG or HbA1c, and only 4.2% of subjects (5/120) had both increased FPG and HbA1c. The use of HbA1c as a criterion for MetS detection was analyzed by McNemar test in which the Chi-square (χ^2^) in MetS-positive group was 38.1 with significance at α = 0.05 and the phi coefficient (ϕ) was 0.591 (p < 0.001), representing a moderate relationship. A stronger relationship was observed in MetS-negative group, in which the Chi-square (χ^2^) was 87.0 and the phi coefficient (ϕ) was 0.851 (p < 0.001). These results demonstrated that the proportion of subjects to be detected with MetS was increased by using HbA1c criterion when compared to FPG criterion.

**Table 4:** **Cross-tabulation contingency table showing FPG and HbA1c in MetS-positive and MetS-negative groups**

			Fasting plasma glucose		
			Yes	No	Total
MetS-positive group	HbA1c	Yes	58	41	99
		No	1	9	10
		Total	59	50	109
MetS-negative group	HbA1c	Yes	5	90	95
		No	1	24	25
		Total	6	114	120

Yes: Fasting plasma glucose or HbA1c above the cutoff and met with MetS criterion.

No: Fasting plasma glucose or HbA1c below the cutoff for MetS criterion.

Our intra-class correlation analysis indicated that the Cronbach’s alpha (α) between FPG and HbA1c was moderately high in MetS-positive group, α =0.725 (95% CI: 0.599 to 0.812, p < 0.001) and in MetS-negative group, α =0.576 (95% CI: 0.391 to 0.704, p < 0.001). The percentage of agreement was 90.7% with the kappa coefficient, κ =0.616 between FPG and HbA1c in those 65 subjects with FPG ≧5.6 mmol/L. However, the percentage of agreement between FPG and HbA1c in MetS-positive group (n =109) was only 61.5% (κ =0.079). This result might be due to the large discrepancy between the prevalence of corresponding criterion in the group (i.e., 59 out of 109 MetS-positive subjects met FPG criterion but 99 of them met the HbA1c criterion).

The diagnostic property of HbA1c at the level of ≧5.7% for MetS diagnosis was evaluated by receiver operating characteristics (ROC) analysis. The area under ROC curve of HbA1c for detecting subjects with MetS relative to FPG was 0.807 (95% CI: 0.727 to 0.887), which yielded a sensitivity level of 98.3%, specificity of 18%, positive predictive value (PPV) of 58.6%, and a negative predictive value (NPV) of 90.0%. When HbA1c cutoff was used to test the presence of MetS, it yielded a sensitivity of 90.8%, specificity of 20.8%, PPV of 51.0%, and NPV of 71.4%. Similar diagnostic properties as indicated by the areas under ROC curve were obtained by using FPG and HbA1c in MetS-positive (0.778 for FPG and 0.736 for HbA1c).

According to the diagnostic criteria of the consensus statement released by the working group report of International Expert Committee in 2009 [20], individuals who have FPG less than 5.6 mmol/L or HbA1c less than 5.7% are defined to be normal. FPG between 5.6 to 6.9 mmol/L or HbA1c between 5.7-6.4% are at pre-diabetic state and with FPG above 7.0 mmol/L or HbA1c ≧6.5% are diagnosed with diabetes [20]. At the beginning of the study, 11 diabetic subjects with FPG ≧7.0 mmol/L were excluded. By considering the level of HbA1c, 47 subjects would have been diagnosed with diabetes (38 from MetS-positive group and 9 from MetS-negative group) which were mid-diagnosed based on the measurement of FPG.

## Discussion

In the present study, the prevalence of metabolic syndrome (MetS) was observed to be increased by 13% after adopting hemoglobin A1c (HbA1c) as the hyperglycemic criterion in the diagnosis of MetS when compared to the use of fasting plasma glucose (FPG) with a good agreement (90.7%, κ = 0.62). These results are in agreement with previous studies reported the increased prevalence of MetS when adopting HbA1c instead of FPG in MetS diagnosis [21, 22]. Nonetheless, it is worth to note that there were also controversial results demonstrated that the use of HbA1c might result in a decreased prevalence of MetS. The prevalence of MetS was reported to be decreased by 4-7% in the study that examined the US National Health and Nutrition Examination Survey data and a Korean cross-sectional study [23, 24]. According to our present data, thirty more participants would be diagnosed with MetS after including HbA1c as a supplementary hyperglycemic criterion. Of note, these thirty participants included 10 participants aged below or equal to sixty years and 20 participants aged above sixty years. If categorized by gender, there were 19 men and 11 women among these thirty participants. Our coarse observations suggested that the adoption of HbA1c hyperglycemic criterion seemed to be more responsive in the male participants of age above sixty. Nevertheless, the observed discrepancy between our present findings and previous studies might be attributed to the differences in study design (i.e., case control study vs. randomized study), age and gender distribution of the subjects, sample size, ethnicity, and pre-analytical or analytical factors which might have affected the measurements of HbA1c and FPG.

Among the Hong Kong Chinese participants in this study, it was observed that the most prevalent MetS components observed were high blood pressure and central obesity, which were similar to the previous observations reported by studies conducted in Italy and Korea [22, 23]. The incidence of hypertension appeared to be higher in the participants aged above 60 particularly those male. It was not surprising that blood pressure was elevated with advanced age due to the fact that arterial stiffness is increased and insulin resistance is common in elderly which generally impair the vaso-dilating function of blood vessels. Besides, Kim and colleagues have demonstrated that the brachial-ankle pulse wave velocity (baPWV), a measurement of arterial stiffness, in male was markedly higher than that in female, which might help to explain the currently observed higher incidence of high blood pressure in men compared to female [23]. In regard to the prevalence of central obesity as measured by waist circumference, it was observed to be more commonly seen in our female participants. It has been proposed that neuro-hormonal dysregulation characterized by the activation of stress-related hormones and age-related decline in sex hormone and growth hormone especially after menopause might lead to increase in deposition of visceral adiposity causing insulin resistance and thus enhances the development of MetS [25]. Indeed, ample amount of evidence have illustrated that the risk factors of the development of MetS were inter-correlated and insulin resistance plays an important role in the pathophysiologic process of the development of MetS. Therefore, once insulin resistance occurs, accumulation of fatty acids might follow which results in the consequence of accelerating other risk factors including hypertriglyceridemia, reduced high-density lipoprotein cholesterol, and hyperglycemia [26].

In the present study, the correlations observed between HbA1c and FPG in all participants and MetS-positive group were moderate, which are consistent with the previous reports [27]. The observed moderate degree of correlation relationship between HbA1c and FPG might be attributed to the differences in the amount of glycosylation that is known to be varied from individual-to-individual [28]. Other possible explanations included change in intra-erythrocyte environment, difference in degree of glycemic control, heterogeneity in the lifespan of red cell, and racial difference [28–30]. Besides, rather high variability of fasting plasma glucose might also result in moderate correlations observed between HbA1c and fasting plasma glucose [31].

Regarding the diagnostic property comparison of HbA1c to fasting plasma glucose in the detection of MetS, the area under the ROC curve in the present study was 0.807 (95% CI: 0.727 to 0.887) which was found to be similar to previous reports that demonstrated moderate-to-high property by adopting HbA1c hyperglycemic criterion, in which the reported area under ROC curve was 0.678 in Succurro *et al*. study and 0.895 in van’t Riet *et al*. study [22, 27]. Based on our results, the areas under the ROC curves by using fasting plasma glucose and HbA1c in MetS diagnosis were 0.778 and 0.736, respectively, suggesting that similar diagnostic values were obtained from fasting plasma glucose and HbA1c for MetS detection. Nonetheless, although HbA1c seemed not offering a higher diagnostic value when compared to fasting plasma glucose, we interpreted that the measurement of HbA1c indeed provided additional information to enhance the detection of hyperglycemic episode in MetS detection. According to the consensus statement of the American Diabetes Association in 2010 [32], an individual having HbA1c at range of 5.7% to 6.4% would be classified as pre-diabetic whereas HbA1c greater than 6.4% would be diagnosed with diabetes mellitus. Of note, 11 participants in MetS-positive group were found to have fasting plasma glucose greater than 7.0 mmol/L at the beginning of this study and these subjects were excluded for data analyses. Thus theoretically, there should have no known diabetic cases in the subject pool being examined in the present study (in which the diagnosis of diabetes is based on fasting plasma glucose). However, we observed that 47 of our included 229 participants (21%) were found having HbA1c level greater than 6.4%, who would have been diagnosed with diabetes mellitus according to the criteria of the American Diabetes Association [32]. These hidden diabetic individuals, whose fasting plasma glucose was normal but HbA1c was elevated, would not be revealed if blood glucose was solely used as the hyperglycemic criterion in the detection process.

Metabolic syndrome (MetS) does not refer to the diagnosis of a disease status but is rather taken as a “sign” to alert individuals who have metabolic abnormalities which are closely associated with the development of diabetes mellitus and cardiovascular diseases [26]. Most importantly, the risk factors and diagnostic components of MetS are readily to be improved and reversed by lifestyle modification (i.e., diet and exercise) in order to delay or prevent the onset of diabetes mellitus and cardiovascular diseases. According to the findings of the present study, HbA1c, which is the glycated form of hemoglobin that reflects the average glucose content in the blood in previous two to three months, together with fasting plasma glucose appeared to be an appropriate hyperglycemic criterion in identifying MetS with an improved detection and in revealing hidden diabetic cases that failed to be detected by solely fasting plasma glucose. Notably, the present study might have some limitations. First, the samples were not randomly obtained from the entire population but from a volunteer-based manner with our adopted case-control study design, which might have selection bias and reduction of generalizability. Besides, similar to the cross-sectional epidemiologic studies, the present study did not have further continuous evaluations in tracking the development of diseases such as diabetes and cardiovascular diseases. In conclusion, the results of this study support the feasibility of the use of hemoglobin A1c (HbA1c) together with fasting plasma glucose (FPG) as the hyperglycemic criteria in the detection of MetS. Although similar diagnostic properties were observed by using HbA1c and fasting plasma glucose in the diagnosis of MetS, the measurement of HbA1c provides additional value in revealing the hidden diabetic cases which were not detected with the sole examination of fasting plasma glucose.
